# Improvement of Omega-3 Docosahexaenoic Acid Production by Marine Dinoflagellate *Crypthecodinium cohnii* Using Rapeseed Meal Hydrolysate and Waste Molasses as Feedstock

**DOI:** 10.1371/journal.pone.0125368

**Published:** 2015-05-05

**Authors:** Yangmin Gong, Jiao Liu, Mulan Jiang, Zhuo Liang, Hu Jin, Xiaojia Hu, Xia Wan, Chuanjiong Hu

**Affiliations:** 1 Key Laboratory of Biology and Genetic Improvement of Oil Crops, Ministry of Agriculture, Oil Crops Research Institute of Chinese Academy of Agricultural Sciences, Wuhan, P.R. China; 2 Hubei Key laboratory of Lipid Chemistry and Nutrition, Oil Crops Research Institute of Chinese Academy of Agricultural Sciences, Wuhan, P.R. China; University of Huddersfield, UNITED KINGDOM

## Abstract

Rapeseed meal and waste molasses are two important agro-industrial by-products which are produced in large quantities. In this study, solid state fermentation and fungal autolysis were performed to produce rapeseed meal hydrolysate (RMH) using fungal strains of *Aspergillus oryzae*, *Penicillium oxalicum* and *Neurospora crassa*. The hydrolysate was used as fermentation feedstock for heterotrophic growth of microalga *Crypthecodinium cohnii* that produce docosahexaenoic acid (DHA). The addition of waste molasses as a supplementary carbon source greatly increased the biomass and DHA yield. In the batch fermentations using media composed of diluted RMH (7%) and 1-9% waste molasses, the highest biomass concentration and DHA yield reached 3.43 g/L and 8.72 mg/L, respectively. The algal biomass produced from RMH and molasses medium also had a high percentage of DHA (22-34%) in total fatty acids similar to that of commercial algal biomass. RMH was shown to be rich in nitrogen supply comparable to the commercial nitrogen feedstock like yeast extract. Using RMH as sole nitrogen source, waste molasses excelled other carbon sources and produced the highest concentration of biomass. This study suggests that DHA production of the marine dinoflagellate *C*. *cohnii* could be greatly improved by concomitantly using the cheap by-products rapeseed meal hydrolysate and molasses as alternative feedstock.

## Introduction

With the depletion of fossil fuel reserves and the increase of greenhouse gas (GHG) emissions contributed largely by the use of fossil fuels, these unsustainable fuels are needed to be gradually replaced by renewable energy sources [1], including liquid biofuels which have the potential to decrease GHG emissions and to meet the energy demands in the transport sector over the past decades [2,3]. The most common liquid biofuels are biodiesel, which are mainly produced from plant oils, waste cooking oils and animal fats via transesterification [3,4]. On the other hand, development of canola and double-low rapeseed varieties (low erucic acid and glucosinolate contents) enhanced the production of rapeseed as source of edible oils in Canada, China, India and other countries. Production of biodiesel and cooking oils using rapeseed as feedstock had led to the generation of large quantities of rapeseed meal (RSM), a by-product of oil extraction from seeds. The global RSM production has been constantly increasing over the past years due to the growing demand for either biodiesel or edible oils, reaching an estimated production capacity of 68.41 million tons in 2014 [3–5].

RSM is mainly composed of protein, fat, fibres (celluloses, hemicelluloses and lignins), pectin, and minerals with significant amounts of calcium, magnesium, zinc, and copper. Traditionally it has been used as organic fertilizer or as combustible source. RSM can be dried by solvent-toasting and used as feed additive for livestock and poultry due to its high protein content [6]. But efficient utilization of RSM in animal feed industry was limited by the presence of low metabolizable energy, poor palatability, and high levels of fibre and anti-nutritional components such as glucosinolates, phytic acids, and phenolic compounds [7–9]. The high fibre content of RSM has led to digestibility problems for monogastric animals due to lacking of the necessary digestive enzymes. Many attempts, including steam stripping, chemical pre-treatment and enzymatic transformation, have been made to reduce the content of anti-nutritional components and thus to enhance the nutritional value of RSM. However, most of these processes are not efficient enough to remove these chemicals [9,10]. Microbial treatment of RSM was applied to help remove toxic substrates and improve RSM’s digestibility and palatability for its application in animal feeding. Recently, it was developed to be an inexpensive, eco-friendly substrate that supports the growth of some microorganisms in both solid-state and submerged fermentations [11]. Generally, high contents of protein, carbohydrates and minerals existed in RSM cannot be assimilated by the majority of microorganisms such as industrial bacteria, yeasts and microalgae. However, these nutrients could be made accessible for microorganisms when RSM was treated through a short-term solid state fungal fermentation followed by enzymatic hydrolysis [12,13]. Until now, meals from several oil crops including rapeseed, soybean and sunflower have been used as low-cost raw materials for growing microorganisms to produce value-added compounds of industrial interest, such as organic acids [8,14,15], antibiotics [15–17], long-chain omega-3 fatty acids [18], and various enzymes [19–23]. Bioconversion of the oilseed meals into value-added products is not only a cost-efficient application in animal feeding but also a potential solution to the surplus problems.

Apart from nitrogen, carbon is also required for microbial growth in large quantities. Despite its relatively high cost, glucose is the most commonly used carbon source in microbial fermentation. Some low-cost inorganic and organic carbon sources, such as crude glycerol from biodiesel plant, and starch hydrolysates from starch-rich crops, could be easily available as cheap alternatives to glucose [24,25]. Waste molasses is a by-product of sugar industry, consisting of up to 50% (w/w) total sugars (glucose, sucrose and fructose), suspended colloids, minerals, vitamins and other components [26]. The annual output of waste molasses in China is about 400 million tons, but most of the molasses are simply abandoned or used for animal feeding [27]. It was recently reported that the dumping of molasses into the Jasilco Reservoir, Mexico, had caused several mass deaths of fish and other aquatic animals. Over the past decades, waste molasses has been developed as a cheap carbon source for production of numerous industrially important chemicals, such as lactic acid [28], citric acid [29], polysaccharide [30], microalgal oils [27], and astaxanthin [31].

**Fig 1:**
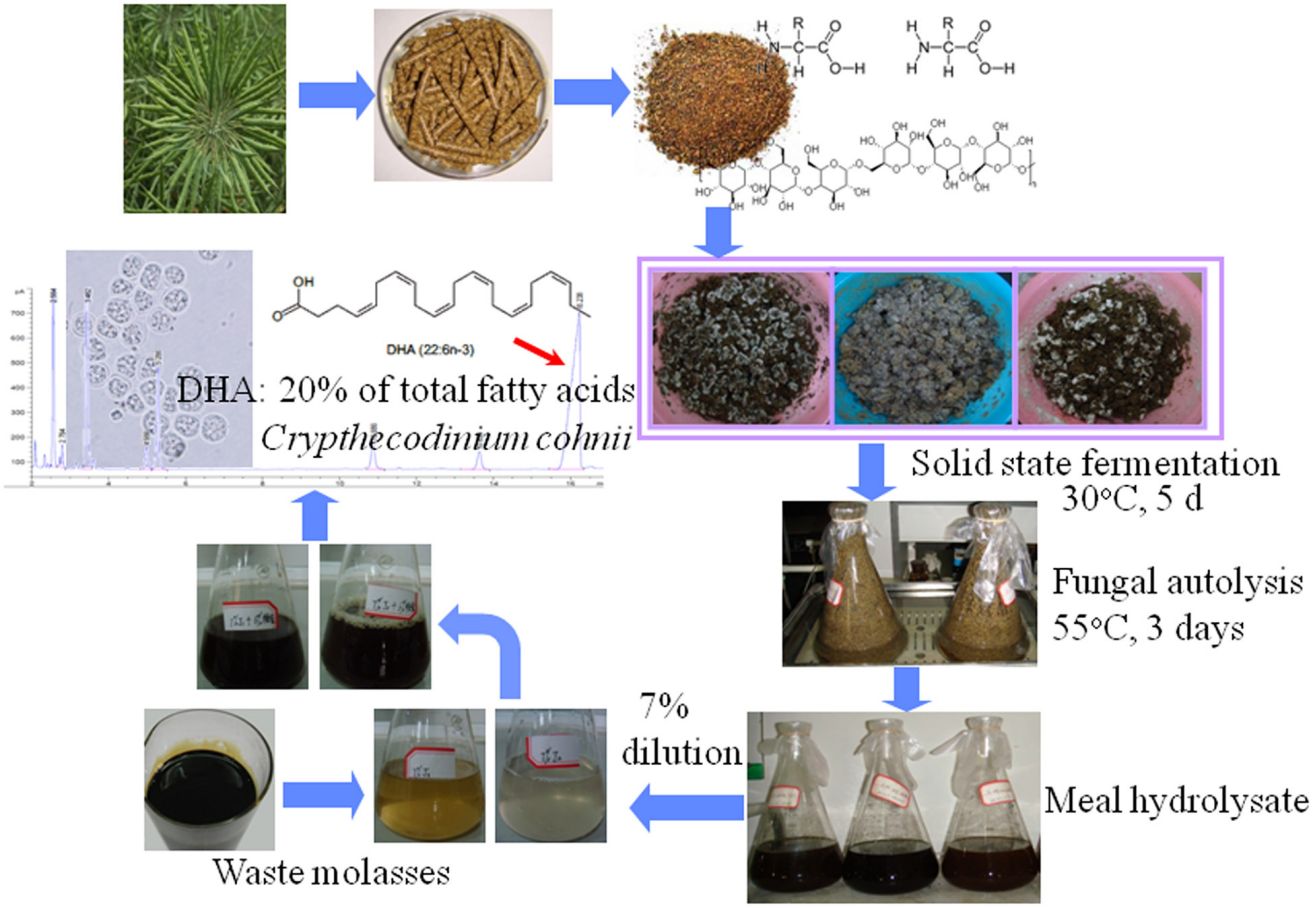
Schematic diagram of the novel DHA production process using by-products for microalgal fermentation. The rapeseed meal hydrolysate was obtained through the treatment of solid state fermentation followed by fungal autolysis. It was used as organic nitrogen source, and the hydrolyzed waste molasses was used as organic carbon source.

Although either RSM or waste molasses alone could be used for cultivation of microorganisms to produce value-added products, simultaneous utilization of these two industrial and agricultural by-products for production of high-value products has not been reported so far. Here, we report a biotechnological application of both RSM and waste molasses as fermentation feedstock to produce omega-3 docosahexaenoic acid (DHA, 22:6 n-3). DHA is one of the most important very long-chain polyunsaturated fatty acids (PUFAs), having many health benefits such as reducing the risks of cardiovascular diseases, cancer, and rheumatoid arthritis, alleviation of depression symptoms and post natal depression, and contributing to the immune-modulatory effects [32,33]. DHA also has important physiological role in healthy development of the fetal brain and retina and thus is commonly included in infant formula and other infant related food products [34,35]. Marine fish oil has been the most common source of DHA in the last decades but it is not able to meet the increasing demand of DHA for human consumption due to the depletion of wild fish stocks and potential pollution of marine environment [36]. Efforts to explore alternative sources of DHA have been made in the last decade, including generation of transgenic oilseed plants [37] and large-scale production of DHA-producing microalgae and protists [38]. *Crypthecodinium cohnii*, a marine heterotrophic dinoflagellate, has been used for industrial production of DHA because it can accumulate DHA at high concentrations within the cells [38]. Glucose and yeast extract are the principle carbon and nitrogen sources needed for large-scale production of DHA through *C*. *cohnii* fermentation. In this study, the alternative fermentation medium was developed, using waste rapeseed meal and molasses, to produce DHA at a level comparable to that obtained in commercial medium containing expensive glucose and yeast extract. This process was summarized in Fig 1. The overall cost of DHA production might be greatly reduced through fermentation of *C*. *cohnii* on this cheap and eco-friendly medium.

## Materials and Methods

### Microorganisms, media and Electron microscopy

Fungal strains of *Aspergillus oryzae* LZ01, *Aspergillus awamori* LZ02, *Penicillium oxalicum* J1 and *Neurospora crassa* J2 were isolated and stored in the laboratory. Of them, three strains were used in solid state fermentations of rapeseed meal. Stock cultures were maintained on potato dextrose agar (PDA) media at 28°C for 5 d, stored at 4°C and transferred every 2–3 months. Spores grown in PDA or wheat bran medium were used as inocula for the solid fermentation. Inoculum size to all fermentations was controlled at about 1–2 million spores to each gram of rapeseed meal on the basis of dry weight.

The microalga *C*. *cohnii* ATCC30772 was used to evaluate the microbial feedstock derived from rapeseed meal throughout this study. *C*. *cohnii* ATCC30772 was purchased from American Typical Culture Collection (USA) and maintained by sub-cultivation on a complex medium composed of 2 g/L yeast extract, 9 g/L glucose, and 25 g/L sea salt. For electron microscopy analysis, shake-flask cultures were grown in flasks (500 mL) containing 200 mL glucose-based medium composed of (per liter) glucose (9g), yeast extract (2g), and sea salts (25g), or RSM-based medium composed of (per liter) RSM hydrolysate (7% vol/vol), molasses (6% vol/vol), and sea salts (25g). These cultures were inoculated (5% vol/vol) with the same starter cultures and grown at 22°C for 7 d with shaking at 180 rpm. The *C*. *cohnii* cultures were sampled at 7 d for electron microscopy preparation. Transmission electron microscope (TEM) observation was performed on fixed material that was prepared for routine examination. The samples of *C*. *cohnii* were fixed in 2.5% glutaraldehyde solution in 0.1 M phosphate buffer (pH 7.2) overnight at 4°C, rinsed four times in 0.1 M phosphate buffer and post-fixed in 1% osmium tetroxide in 0.1 M phosphate buffer for 1 h at 4°C. The fixed samples were dehydrated gradually with ethanol, embedded in Spurr’s resin and sectioned on a Lecia Ultracut UCT ultramicrotome equipped with diamond knives. The sections were stained with uranyl acetate and lead citrate and observed under a transmission electron microscope (HITACHI, H-7000FA).

### Cellulolytic activity test

Four fungal strains, namely *Aspergillus oryzae* LZ01, *Aspergillus awamori* LZ02, *Penicillium oxalicum* J1 and *Neurospora crassa* J2, were first inoculated on wheat bran medium (50 g/L bran and 15 g/L agar), and incubated at 28°C for 3 d. To evaluate the cellulolytic activity of extracellular enzymes, approximately equal amounts of spores of these fungal strains were grown on CMC medium composed of 1% (wt/vol) sodium carboxymethyl cellulose (CMC-Na) and 1.4% (wt/vol) agar at 28°C for 3 d. Congo red staining was conducted as described by Teather and Wood [39].

### Rapeseed meal and solid state fungal pre-treatment followed by fungal autolysis

Rapeseed meal was purchased from the Grain Bureau of Jiangxia District, Wuhan, China. The rapeseed meal was kept in air-tight plastic containers and stored at room temperature. Solid state fermentation of rapeseed meal was carried out as described by Wang et al. [12]. Briefly, a certain amount of rapeseed meal was firstly moistened with the required amount of tap water to obtain 65% moisture content in a 1 L bottle and then sterilized at 121°C for 45 min. The sterilized meal was allowed to cool down to room temperature and then inoculated with the desired population of fungal spores (approximately 10^6^ spore/g rapeseed meal). For spore preparation, each strain of *A*. *oryzae*, *N*. *crassa* or *P*. *oxalicum* was inoculated on wheat bran medium composed of 50 g/L bran and 15 g/L agar, and incubated at 28°C for 5 d. The spores were washed with sterile glass beads from the plates and then diluted into known volume of sterile distilled water before inoculation. The meal inoculated with spores was mixed and incubated at 30°C for 4 d. After the solid fermentation, autolysis of the fungi was initiated by mixing two-fold volume of distilled water with the fermented solids to obtain approximately 50–60 g/L solid concentration. The mixture was homogenized using a kitchen blender and then incubated at 55°C for 3 d in a baffled flask. The generated autolysate was centrifuged at 12,000g for 10 min to remove solid residues. The supernatant was filtered through a 0.22 μm filter and kept at 4°C until use.

### Pretreatment of crude molasses

Crude waste molasses was obtained from the Jinqianwan Molasses Co., Ltd (Liuzhou, Guangxi Province, China). It was diluted with distilled water at a ratio of 1:1–1.5 (v/v). The pH of the generated solution was adjusted to 3.0 with 4 M sulfuric acid. The acidic solution was then heated to 100°C for 1 h or 60°C for 2 h, allowed to cool down to room temperature and incubated overnight. The residue was removed by centrifugation at 8,000 g for 15 min and the supernatant was collected. The pH of the supernatant was adjusted with 10 M NaOH to 6.5, which can be used for medium preparation [27].

### Microalgal oil production using the RSM-based media

To make starter cultures, *C*. *cohnii* ATCC30772 was incubated statically at 25°C for 4 d in 250-mL flasks containing 100 mL medium (2 g/L yeast extract, 9 g/L glucose, and 25 g/L sea salt). Starter cultures were harvested by centrifugation (2500 ×g, 10 min), and washed twice with sterile distilled water prior to flask fermentation. These static cultures were then used to inoculate shake-flask cultures at a ratio of 1:10 (v/v %). Shake-flask cultures were grown in 500-ml flasks containing 200 ml of bio-medium composed of 7% RMH and 25 g/L sea salt.

To test the effect of yeast extract on biomass concentration and lipid synthesis, different concentrations of yeast extract (0, 0.4, 1, 1.8, 2.8, 4.0 g/L) were added into the above described bio-medium. To evaluate the effect of waste molasses on the enhancement of biomass and lipid production, different concentrations of waste molasses (0, 1%, 3%, 6%, 9%, v/v) were added. For the comparison of biomass and DHA yields of *C*. *cohnii* grown on different carbon sources, 2.97 g/L glucose, 2.97 g/L galactose, 4.06 g/L sodium acetate, 2.89 mL/L anhydrous ethanol and 49.5 mL/L waste molasses was added into the same bio-medium individually. Each of these chemicals contains 0.1 mol/L carbon in the RMH-based media. All cultures described as above were grown at 22°C for 7 d with shaking at 180 rpm, and collected for determination of biomass concentration, fatty acid composition and DHA content.

### Analytical methods

Free amino nitrogen (FAN) concentration in the rapeseed meal during solid state fermentation was determined by the ninhydrin colorimetric method [40]. The concentration of inorganic phosphorus was analyzed by the ammonium molybdate spectrometric method [41].

Algal biomass concentration was determined by centrifugation and lyophilization as described previously [42]. Samples of approximately 50 mL cultures were centrifuged at 1,500 g for 5 min. The cell pellet was washed in 20 mL double distilled water, lyophilized, and weighed in order to determine the biomass concentration. For lipid extraction, freeze-dried cells (100 mg) were weighed accurately into a 10 mL-centrifuge tube. Approximately 3 mL chloroform:methanol (2:1, v/v) solution containing 0.5 mg/mL butylated hydroxytoluene and 1.0 mg/mL heptadecanoic acid was added and the centrifuge tube was shaken gently overnight. The solution with the extracted lipids was separated from the cell debris by centrifugation at 1,500 g for 5 min. The supernatant contained the lipid extract. Methylated fatty acids were prepared from the lipid extract by trans-methylation with methanol-acetyl chloride, extracted with hexane, and analyzed by gas chromatography as previously described [43]. Fatty acid methyl esters were identified by chromatographic comparison with authentic standards (Sigma). Individual fatty acid was quantified from peak area on the chromatogram using heptadecanoic acid as the internal standard.

## Results

### Meal hydrolysate produced by fungal fermentation and autolysis

**Fig 2:**
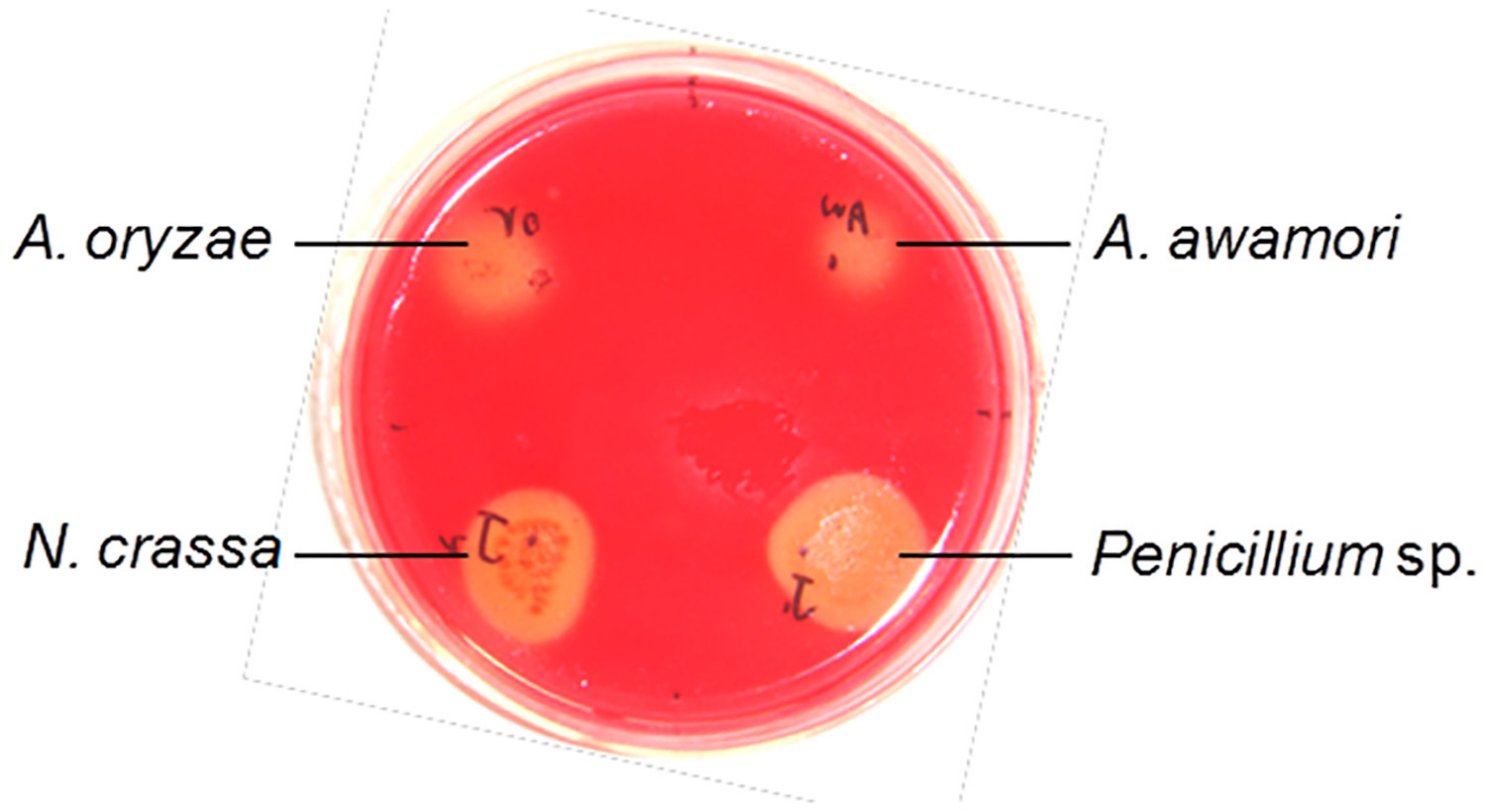
Congo red staining of fungal strains with cellulolytic activity. The fungal strains were grown on agar plate containing 1% (wt/vol) sodium carboxymethyl cellulose (CMC-Na) and 1.4% (wt/vol) agar. CMC hydrolysis was detected by the Congo red staining after being incubated for 3 d.

Solid state fungal pre-treatment followed by fungal autolysis was found to be a good strategy to produce a generic microbial feedstock from rapeseed meal. This approach is effective and cost-efficient compared to other described pre-treatment processes such as commercial enzymes, liquid state fungal pre-treatment and liquid state fungal pre-treatment using fungal enzymatic broth [13]. In this solid state pre-treatment process, RSM could provide all essential nutrients for fungal spore germination and growth. Apart from protein, RSM is also rich in carbohydrates, which could be utilized by fungi through their own hydrolytic enzymes. Based on screening for strains with high activity of glycohydrolysis, four candidate strains *A*. *oryzae* LZ01, *A*. *awamori* LZ02, *P*. *oxalicum* J1 and *N*. *crassa* J2 were selected. All of them were able to grow well on medium containing CMC-Na, or Mannan or Xylan as the sole carbon source. Fig 2 shows the cellulolytic activity of these four strains as revealed by positive Congo-red staining. As strain LZ01 and LZ02 are from same species with close activity, thus strains of LZ01, J1 and J2 were eventually selected for the throughout experiments including solid state fermentation and autolysis.

**Fig 3:**
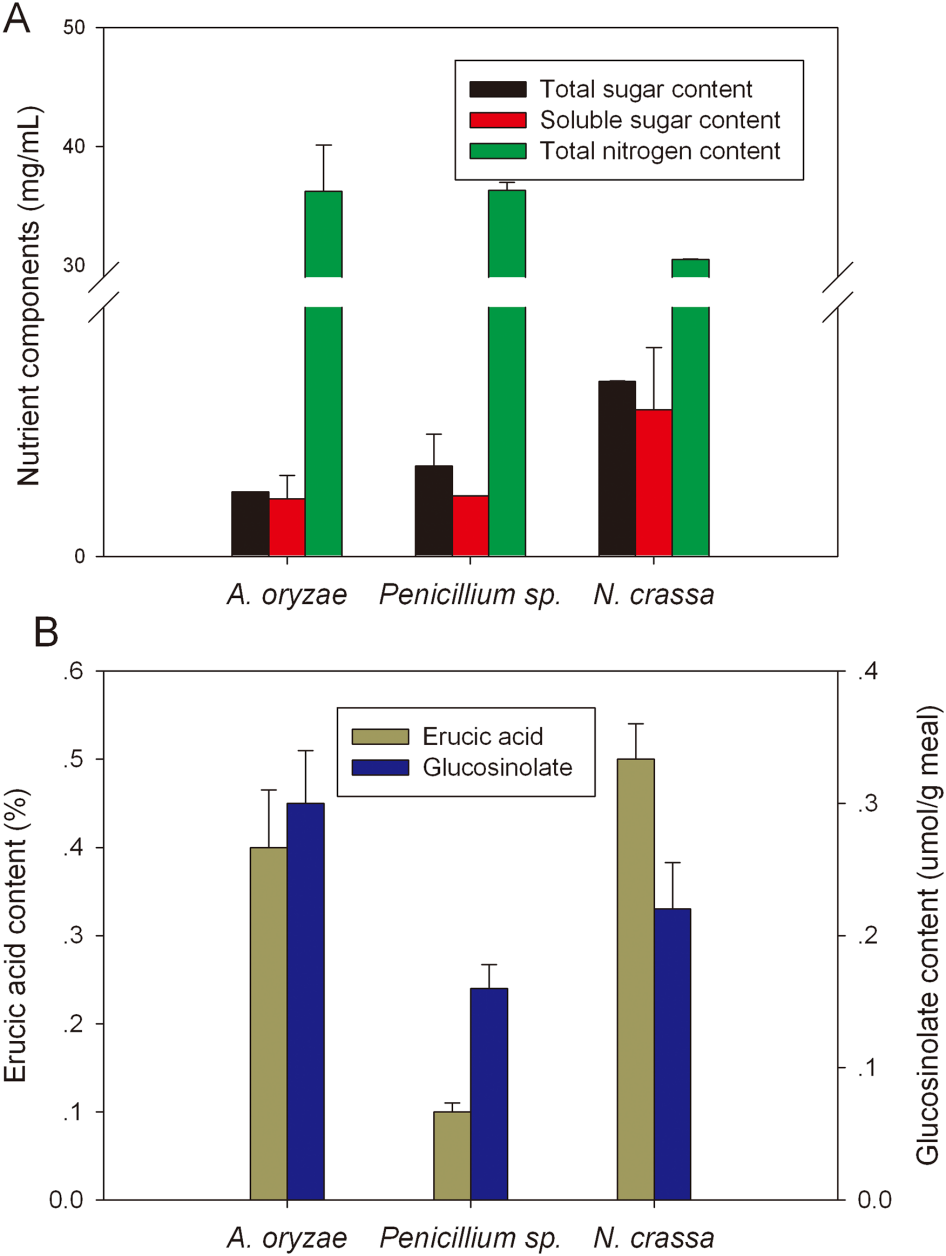
Total nitrogen and sugar contents of the meal hydrolysates. RMHs were obtained by fungal solid state fermentation (pre-treatment) followed by autolysis. Three fungal strains were used for solid state fermentation and autolysis individually. Data are means of three replicates, and error bars show standard deviations.

**Fig 4:**
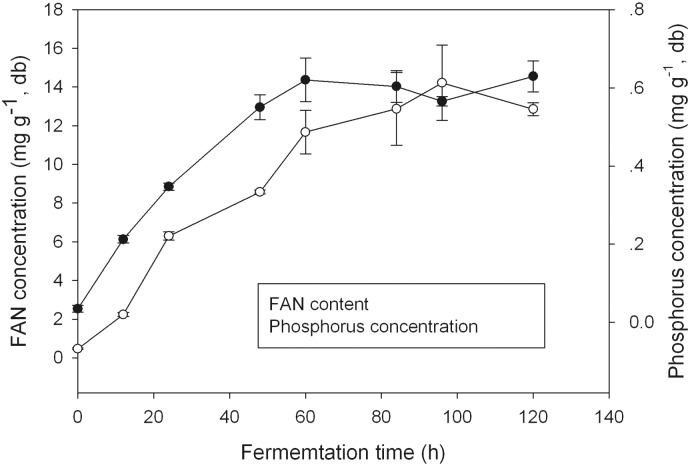
The contents of free amino nitrogen and inorganic phosphorus in *N*. *crassa* derived hydrolysate. The rapeseed meal was fermented with *N*. *crassa* J2 at 30°C and sampled at various time points for analytical experiments; Data are means and standard deviations from triplicate fermentations.

Pre-treatment of RSM using LZ01, J1 or J2 followed by autolysis was performed to produce nutrients-rich hydrolysate. The hydrolysate LZ01, J1 and J2 contained almost equivalent content of total nitrogen, respectively, 36.22, 36.31 and 33.49 mg/mL. Each hydrolysate also contained quite high amount of soluble sugar; but the J2 hydrolysate contained the highest content of 234.8 mg/L, which was nearly 2.5 times higher than that of other two hydrolysates generated by *A*. *oryzae* LZ01 and *P*. *oxalicum* J1 (Fig 3A). Detoxification of RSM by solid state fermentation with these three strains was also assessed. There were very low levels of erucic acid (0.1–0.5%, w/w) and total glucosinolates (0.16–0.30 μmol/g meal) detected in the RSM hydrolysates (Fig 3B), while about 2% erucic acid contained in the seed oil and 30 μmol/g of total glucosinolates in the seed meal from the commercial canola cultivar [44]. The beneficial effect of microbial fermentation on reduction of anti-nutritional components has been reported previously. Fermentation of RSM with *Rhizopus oligosporus* for 10 days resulted in the reduction by 43.1% for glucosinolates, 34% for thiooxazolidones and 42.4% for phytic acid, respectively [7]. During the solid state fermentation, we observed the continuous release of nitrogen and phosphorus in the fermenting meal. An obvious linear increase in free amino nitrogen (FAN) content was observed during the first 60 h fermentation period; with the maximum content of 11.67 mg/g (db) at the time point of 60 h. The increasing trend paused during the subsequent 60 hours of fermentation, the FAN content remained as high as the maximum content over the time (Fig 4). This might indicate the accumulation of protease in the fermenting meal, as protease played role in formation of FAN [45]. In contrast, a linear increase in phosphorus content was observed within the longer span of time, with the maximum phosphorus content of 0.63 mg/g (db) at time point of 100 h. The continuous increase in inorganic phosphorus content might be attributed to the production of phytase and phosphatase in the fermented meal [12]. From these results it can be concluded that microbial treatment of RSM not only increased the release of accessible nitrogen source but also resulted in degradation of toxicants in meal.

### Evaluation of meal hydrolysate as bio-medium for production of microbial oils

**Fig 5:**
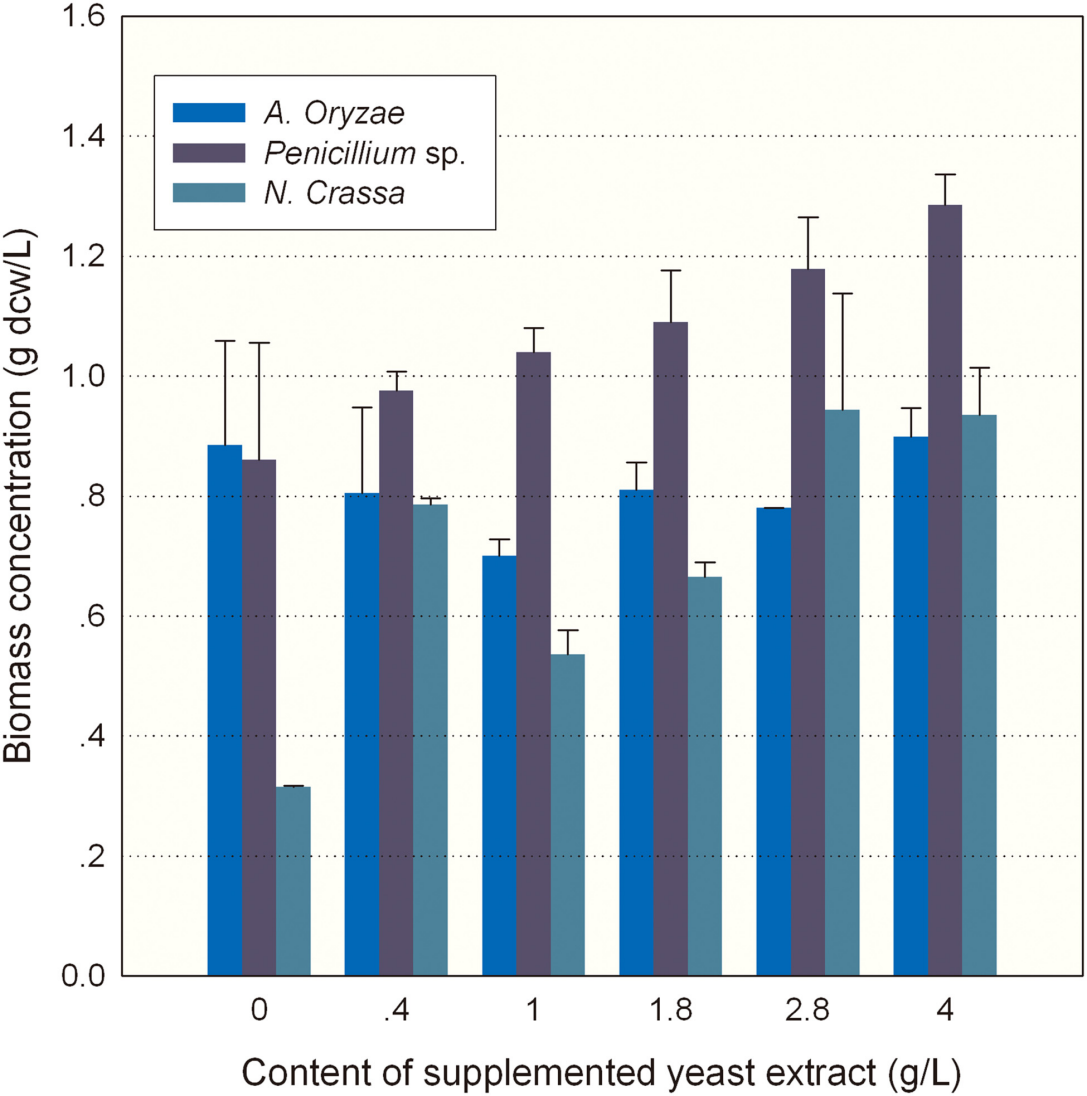
Biomass concentration of *C*. *cohnii* grown on RMH supplemented with yeast extract. The meal hydrolysates produced by individual fungal stains were supplemented with different levels of yeast extract. Data of biomass concentration are means of three replicates, and error bars show standard deviation.

We tested the feasibility of using meal hydrolysate as a sole nutrient supplement for production of microbial oils by growing seven different oleaginous strains. It was found that a basal medium composed of sea-salt and the RMH diluted by 2 to 7-fold could well support the growth of oleaginous microbes tested, including four yeast strains of *Trichosporon fermentans*, *Lipomyces kononenkoae*, *Lipomyces starkeyi* and *Rhodotorula glutinis*, two filamentous fungi of *Rhizopus stolonifer* and *Mortierella* sp., and one microalgal strain *C*. *cohnii* ATCC30772. However, the biomass concentrations of these oleaginous microorganisms in medium containing RMH alone were much lower than that achieved on YPD medium. This prompted us to investigate whether additional nitrogen or carbon source is needed to supplement the RSH media. Thus the DHA-producing heterotrophic microalga *C*. *cohnii* was selected and incubated in eighteen media made of the 7-fold diluted RMH (generated by LZ01, J1 or J2) supplemented with yeast extract ranging from 0.4 g/L to 4.0 g/L. As the amount of supplemented yeast extract increased, there was no obvious increase in biomass concentration of *C*. *cohnii* grown in LZ01-derived RMH, but increases were observed in the J1- or J2-RMH (Fig 5). The maximum biomass concentration among the three hydrolysates was 1.28 g/L, which was reached in the medium made of J1-RMH supplemented with 4.0 g/L yeast extract. Substantially, the effect of yeast extract supplementation was insignificant in increasing the algal biomass concentration. This thus implied that nitrogen contained in the RSM hydrolysate was sufficient enough to support algal growth.

Table 1 shows the total fatty acid content and yield, DHA yield and unsaturated fatty acid yield for *C*. *cohnii* cells grown on various RMH supplemented with different amounts of yeast extract. With the increase of yeast extract level up to 4 g/L, the total fatty acid content and yield did not show much increase in all hydrolysates. However, supplementation of yeast extract into the J2-derived RMH led to about 2–3 fold increase in DHA yield and unsaturated fatty acid yield (Table 1, in bold), compared with that grown on LZ01- or J1-RMH. This result indicated that the nitrogen content in *N*. *crassa*-derived RMH (Fig 3A) may not enhance the accumulation of DHA or other PUFAs and the additional nitrogen may be needed. Together, RMH are nutritious enough in supporting the algal growth.

**Table 1:** Effect of supplemented yeast extract on lipid production by *C*. *cohnii* on individual RMH medium.

RMH medium derived by	Yeast extract content (%)	TFA^a^ content(mg/g)	TFA yield (mg/L)	DHA yield (mg/L)	UFA^b^ yield (mg/L)
*A*. *oryzae*	0.0	35.10±2.47	31.06±7.01	5.68±2.08	8.42±2.60
	0.4	29.64±2.02	23.86±3.83	4.66±0.11	6.09±0.49
	1.0	36.74±9.06	25.72±2.43	5.37±0.73	7.41±0.28
	1.8	33.77±7.27	27.35±2.84	5.98±0.99	7.61±1.42
	2.8	36.67±0.80	28.60±3.71	6.56±1.37	8.55±0.53
	4.0	33.37±0.17	27.51±2.90	6.79±0.13	8.54±0.21
*Penicillium* sp.	0.0	37.80±0.86	40.54±3.55	8.69±2.19	12.87±0.20
	0.4	40.59±3.24	52.98±4.12	9.18±0.32	16.35±1.14
	1.0	38.76±6.03	40.31±2.64	7.90±1.17	11.08±0.28
	1.8	41.11±5.48	44.77±2.74	9.37±0.49	11.96±0.80
	2.8	36.24±3.91	42.69±2.52	10.13±1.35	13.22±2.11
	4.0	41.15±1.72	52.88±0.33	10.59±0.12	13.89±0.69
*N*. *crassa*	0.0	73.72±6.08	23.22±1.33	2.78±0.31	5.66±0.14
	0.4	70.87±0.80	74.29±0.31	5.59±0.21	14.26±2.89
	1.0	52.41±1.02	66.58±4.03	6.02±2.03	12.41±0.22
	1.8	55.09±1.76	59.52±1.09	9.60±0.50	15.06±0.57
	2.8	52.72±0.32	88.31±0.82	9.90±0.04	16.58±0.06
	4.0	58.64±1.21	54.83±3.93	10.13±0.53	14.66±0.84

^a^ TFA, total fatty acids;

^b^ UFA, unsaturated fatty acids

### Effect of waste molasses as a supplement on biomass and lipid production

**Fig 6:**
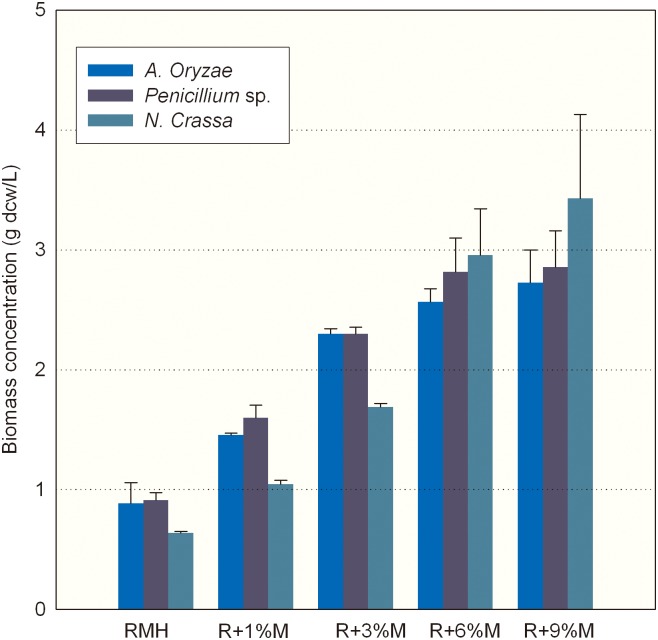
Biomass concentration of *C*. *cohnii* grown on individual RMH supplemented with waste molasses. The meal hydrolysates produced by individual fungal stains were supplemented with different levels of molasses. Data of biomass concentration are means of three replicates, and error bars show standard deviation.

In addition to a complex and low-cost nitrogen source like RMH, an inexpensive carbon source must also be provided to the fermentation medium to support rapid growth of algal cells. To discover cheap carbon source for algal biomass production, we attempted to use several agricultural or industrial by-products as substrate. It was found that the biomass of *C*. *cohnii* could not be further increased by the addition of sucrose waste stream, starch hydrolysates or biodiesel-derived crude glycerol in all three meal hydrolysates, indicating that these low-cost substrates are not the appropriate carbon sources for the growth of *C*. *cohnii*. It has been demonstrated that waste molasses could serve as fermentable carbon source for oil production by the microalga *Chlorella* sp. [27,31]. To investigate the effect of waste molasses supplementation on biomass concentration, the individual meal hydrolysate was used as basal medium (composed of mainly nitrogen source) and various amounts of molasses were supplemented. As shown in Fig 6, upon the increase of supplemented molasses, dramatic increases in biomass concentration of *C*. *cohnii* were observed in all three RMH media with the maximum biomass concentration reached 3.43 g/L in J2-RMH supplemented with 9% molasses. Although RMHs produced by three fungal strains contained certain amounts of soluble sugars, it might not meet the need of high density growth of *C*. *cohnii*. Therefore, the industrial by-product molasses might be an ideal alternative to glucose in increasing the algal biomass.

The effect of molasses on fatty acid content and yield of *C*. *cohnii* was investigated as well (Table 2). The results showed that both total fatty acid content and yield of *C*. *cohnii* were obviously enhanced by molasses supplementation. The maximum total fatty acid yield of 26.93 mg/L was achieved in *N*. *crassa*-produced hydrolysate (J2-RMH) supplemented with 6% molasses. And the yield of DHA and unsaturated fatty acid of *C*. *cohnii* were all elevated when grown in three RMH media (Table 2, in bold). J2-RMH supplemented with 6% of molasses gave rise of the highest yield of DHA (8.72 mg/L) and total unsaturated fatty acid (10.38 mg/L). It was found that addition of 3% molasses into LZ01-RMH could lead to 5-fold increase in total fatty acid content and 7-fold increase in total fatty acid yield, respectively. It suggested that carbon nutrient was quite limited in *A*. *oryzae*-produced hydrolysate. Addition of molasses as supplementary carbon source could remarkably increase both the biomass concentration and DHA yield of *C*. *cohnii*.

**Table 2:** Effect of supplemented waste molasses on lipid production by *C. cohnii* on individual RMH medium.

RMH medium derived by	Molasses content	TFA content (mg/g)	TFA yield (mg/L)	DHA yield (mg/L)	UFA yield (mg/L)
*A*. *oryzae*	1%	5.85±0.12	2.53±0.05	0.57±0.01	0.75±0.03
	3%	24.99±0.43	19.66±0.26	4.40±0.21	5.75±0.21
	6%	24.97±0.73	19.65±1.09	5.16±0.23	5.90±0.45
	9%	23.43±1.61	22.59±1.60	6.19±1.19	7.06±0.68
*Penicillium* sp.	1%	28.61±1.38	13.45±1.17	2.99±0.87	4.77±0.85
	3%	32.09±1.92	21.07±1.02	6.56±0.55	8.00±0.25
	6%	25.79±1.40	21.84±1.88	6.09±0.16	9.23±0.69
	9%	31.58±1.22	25.38±0.74	6.27±0.51	9.10±0.73
*N*. *crassa*	1%	19.83±2.63	6.19±0.53	1.69±0.40	2.73±0.72
	3%	31.83±0.51	15.80±0.99	3.98±0.67	5.26±0.29
	6%	27.73±1.02	26.93±0.88	8.72±0.91	10.38±1.12
	9%	22.02±0.32	23.69±0.70	4.59±0.59	5.73±0.98

^a^ TFA, total fatty acids;

^b^ UFA, unsaturated fatty acids

**Fig 7:**
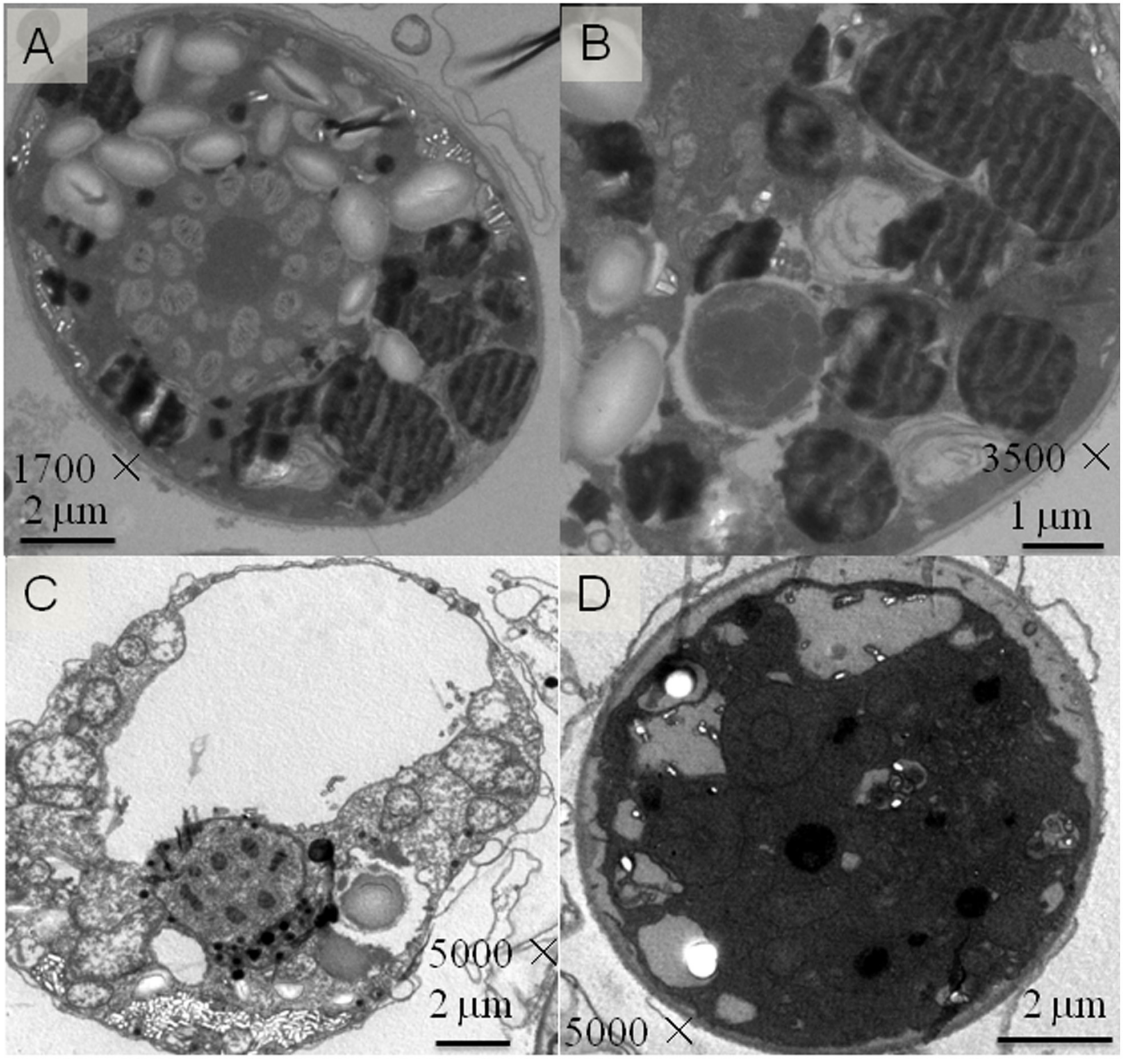
Transmission electron micrograph of *C*. *cohnii* cells. Microalga cells were grown on seawater based GYE containing glucose and yeast extract (**A**, **B**) or RMH-molasses medium composed of the meal hydrolysate and waste molasses (**C**, **D**) for 7 days.

As suggested by the results shown in Fig 5, it was likely that RMH contained higher concentration of available nitrogen in contrast to the conventional glucose/yeast extract (GYE) medium. This may explain why oil body formation was observed primarily in cells grown on the GYE medium but not in cells on the RMH-molasses medium 7 days after incubation (Fig 7). The RMH-molasses medium was likely to have high N-content or N/C ratio. Over the time when algal biomass increased and the available nitrogen supply was consumed, many oil bodies must be formed as RMH-molasses medium was shown to enhance the DHA production (Table 2). The delayed oil drop formation of *C*. *cohnii* cells might be attributed to the existence of relatively high N-content in RMH medium during first several days. It was rationale and necessary to supplement molasses into the RMH medium to properly decrease the N/C ratio to favor the lipid synthesis. Electron microscopic results revealed that the algal cellular organelles were normal in both RMH-molasses and GYE medium, indicating that RMH-molasses medium is as good as GYE in maintaining algal growth and lipid production. It was demonstrated that RMH-molasses medium not only enhanced the production of biomass and DHA but also the total fatty acids. Electron micrograph showed that *C*. *cohnii* grew vitally on RMH-molasses medium as the cells were dominantly small and full of high electron density material in cytosol (Fig 7C and 7D). This is common that environmental change of nitrogen supply affects the cell size of microorganisms including bacteria, yeast and algae [46–48].

### Effect of meal hydrolysate supplemented with different carbon sources for algal DHA production

**Fig 8:**
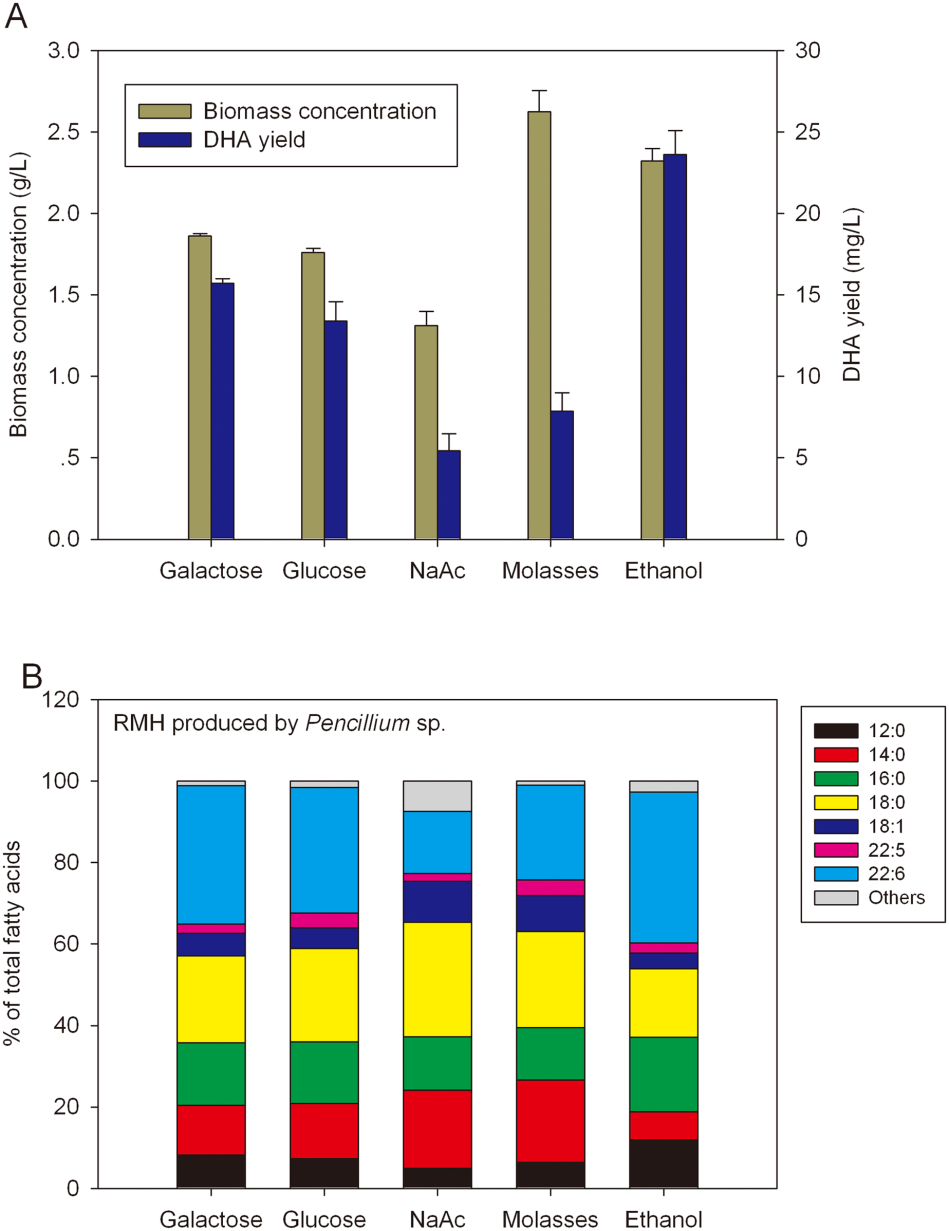
Biomass and lipid production by *C*. *cohnii* grown on RMH supplemented with different carbon sources. (**A**) Effect of supplemented carbon sources on biomass concentration and DHA yield of *C*. *cohnii* grown on basal meal hydrolysate. Data are means of three replicates, and error bars show standard deviation; (**B**) Fatty acid composition of *C*. *cohnii* grown on the *Penicillium* sp.-produced RMH supplemented with different carbon sources.

Given that medium derived from RMH contained enough amount of assimilable FAN (Figs 5 & 6), it was speculated that increase of carbon supply could increase lipid accumulation in *C*. *cohnii* because high N-content or N/C ratio (of RMH) usually favors the increase of cell numbers and delays the process for lipid synthesis. We further tested the conversion efficiency of carbon tozuij oil using various carbon sources that can be readily used by *C*. *cohnii*. Carbohydrate of glucose, galactose, sodium acetate, molasses or ethanol was added at concentration of 0.1 M into RMHs to make the respective RMH-plus media for fermentation. As shown in Fig 8A, upon the addition of individual carbohydrate, both biomass concentration and DHA yield of *C*. *cohnii* were elevated, with the highest increase in biomass concentration occurred clearly in the presence of molasses. Of five carbon sources tested, only molasses and ethanol produced over 2 g/L biomass concentration (Fig 8A). Upon the addition of molasses, algal biomass concentration topped as high as 2.6 g/L dry cell weight (dcw), indicating that molasses could be a cheap alternative to monoses. Interestingly, upon the addition of ethanol, *C*. *cohnii* generated high amount of DHA (23.6 mg/L) and biomass (2.3 g/L), displaying the highest percentage of DHA in total fatty acids (Fig 8B). This indicates that addition of ethanol might somehow favor the algal cell growth and lipid production.

## Discussion

*C*. *cohnii* ATCC30772 is commonly maintained or fermented on GYEmedium which is quite rich in nitrogen. Hence, for the scale-up fermentation point of view, large-volume use of GYE is thought to be very cost-ineffective because commercial yeast extract or peptone is expensive. It is imperative to find out cheap alternatives including rapeseed meal. Evans et al. [49] reported that an oleaginous *Rhodosporidium* sp. was able to accumulate more lipids when organic nitrogen from meal was added. On the other hand, some study suggested that inorganic nitrogen was shown to be more beneficial for biomass accumulation [50]. It was reported that an essential condition for lipid accumulation in oleaginous microorganisms was the use of nitrogen sources in limiting concentrations or low availability [51]. The medium derived from rapeseed is thought to be an ideal nitrogen source although it contains anti-nutrient materials such as erucic acid and glucosinolate. In our study, fungal SSF conversion of the rapeseed meal at room temperature followed by the autolysis at 55°C not only increased the contents of FAN and soluble sugars but also largely reduced the content of anti-nutritional components. Initially, we started with a dozen of fungi and bacteria which have activities of glycohydrolysis against rapeseed meal. Three fungal strains from the genera of *Aspergillus*, *Penicillium* and *Neurospora* and two strains from *Bacillus* were shown to be highly effective in breaking down the insoluble polysaccharides and presumably toxic erucic acid and glucosinolate. Since two *Bacillus* strains are heat-resistant and three fungal strains were very vulnerable to the heating-mediated autolysis, three fungi were thus selected for all experiments.

To our knowledge, this is the first report about the rapeseed meal-derived medium developed for PUFA production through heterotrophic microalga *C*. *cohnii*. The high cell density and high lipid content were achieved by alternative medium made of by-products like rapeseed meal and molasses. Both kinds of by-products have not been well utilized due to lack of cost-efficient approaches and bad management. Our study demonstrates that rapeseed meal can be bio-converted into nutrients-rich hydrolysates which in turn can be used to develop eco-friendly media to nourish PUFA-producing microorganisms. Although this bioconversion process for rapeseed meal is similar to that for yeast lipid production [12], we extend *A*. *oryzae* to several fungal strains that were used for solid state fermentation and production of hydrolytic enzymes, which could release several types of nutrients from rapeseed meal. Additionally, we observed that adding molasses to RSM-based culture of *C*. *cohnii* could enhance microalgal growth and DHA accumulation, and the components contained in molasses exhibited no any inhibitory effect on algal growth. Compared to this beneficial effect on microalgal growth, soap and methanol, the major impurities contained in crude glycerol, were inhibitory to fungal growth [52]. This might be one of the reasons for low biomass and EPA yield when biodiesel-derived crude glycerol was used as alternative carbon source for producing EPA by the fungus *Pythium irregulare* [52]. In this study, concurrent use of waste molasses, another by-product from sugarcane industry, would further decreased the fermentation costs and reduce the release of molasses into the environment. Therefore, RMH and molasses can be the sustainable and cost-efficient alternatives to relatively expensive yeast extracts and monoses. The strategy could be extended to producing other value-added chemicals from cheap agro-industrial by-products.

## Conclusions

RSM hydrolysates developed through fungal solid state fermentation and autolysis contain rich nutrients especially the organic nitrogen source. The production of biomass and omega-3 DHA by *C*. *cohnii* could be improved by using rapeseed meal hydrolysate and waste molasses as alternative feedstock. The eco-green medium made of 7% meal hydrolysate and 1–9% waste molasses could well support the growth of *C*. *cohnii* and produce DHA accounting for 22–34% of total fatty acids. Together, the cheap by-products of rapeseed meal and molasses can be bio-converted into nutritional feedstock for value-added DHA production through algal fermentation.
